# Nuclear and Cytoplasmatic Players in Mitochondria-Related CNS Disorders: Chromatin Modifications and Subcellular Trafficking

**DOI:** 10.3390/biom12050625

**Published:** 2022-04-23

**Authors:** Matteo Gasparotto, Yi-Shin Lee, Alessandra Palazzi, Marcella Vacca, Francesco Filippini

**Affiliations:** 1 Synthetic Biology and Biotechnology Unit, Department of Biology, University of Padua, Via Ugo Bassi 58/B, 35131 Padua, Italy; matteo.gasparotto.1@phd.unipd.it; 2 Institute of Genetics and Biophysics “A. Buzzati Traverso”, CNR, Via Pietro Castellino, 111, 80131 Naples, Italy; yi-shin.lee@unina.it (Y.-S.L.); alessandra.palazzi@igb.cnr.it (A.P.); marcella.vacca@igb.cnr.it (M.V.); 3 Pharmacology Division, Department of Neuroscience, Reproductive and Odontostomatological Sciences, Faculty of Medicine and surgery, University of Naples Federico II, Via Pansini 5, Building 19 (Biological Tower), 80131 Naples, Italy

**Keywords:** mitochondria, chromatin remodeling, subcellular trafficking, autophagy, mitophagy, MeCP2, small GTPase, longin domain, Rab, VAMP, SNARE

## Abstract

Aberrant mitochondrial phenotypes are common to many central nervous system (CNS) disorders, including neurodegenerative and neurodevelopmental diseases. Mitochondrial function and homeostasis depend on proper control of several biological processes such as chromatin remodeling and transcriptional control, post-transcriptional events, vesicle and organelle subcellular trafficking, fusion, and morphogenesis. Mutation or impaired regulation of major players that orchestrate such processes can disrupt cellular and mitochondrial dynamics, contributing to neurological disorders. The first part of this review provides an overview of a functional relationship between chromatin players and mitochondria. Specifically, we relied on specific monogenic CNS disorders which share features with mitochondrial diseases. On the other hand, subcellular trafficking is coordinated directly or indirectly through evolutionarily conserved domains and proteins that regulate the dynamics of membrane compartments and organelles, including mitochondria. Among these “building blocks”, longin domains and small GTPases are involved in autophagy and mitophagy, cell reshaping, and organelle fusion. Impairments in those processes significantly impact CNS as well and are discussed in the second part of the review. Hopefully, in filling the functional gap between the nucleus and cytoplasmic organelles new routes for therapy could be disclosed.

## 1. Introduction

In the past decade, excellent research has unveiled mitochondria to play a pivotal role in several processes other than their established role as “cellular power plants”. Indeed, these organelles are involved in processes underlying the regulation of cell, tissue, organ, and organism life and developmental pathways [1,2,3].

When considering CNS diseases related to mitochondria, first thoughts go to neurodegeneration because of the huge literature provided so far by research on Parkinson’s disease (PD) [4,5]. However, in this review, we focus on the less debated relationships among mitochondria, neurodevelopment, and non-degenerative neurological disorders. We illustrate how impaired mitochondrial function, associated with CNS diseases, may depend on events occurring both in the nucleus and in the cytoplasm, with a particular emphasis on chromatin remodeling and transcriptional control, vesicle biogenesis, fusion, subcellular trafficking, and autophagy. Within such cellular processes, key molecular players can be identified. Those include “building block” families of proteins with conserved domains and functions that often are partners in conserved interaction complexes and pathways. Regulators of chromatin plasticity have been deeply studied and reviewed for their functions in DNA repair, replication and recombination, and transcriptional regulation [6,7]. In contrast, the mutual relationship between nuclear chromatin players and the mitochondria landscape has often been neglected and it is described in this review. Finally, while regulatory distortions in cytoplasmic and nuclear components have often been studied as distinct topics we propose a hypothesis on their interconnection. Unraveling the complexity of the cytoplasmic- nuclear interaction in mitochondria-related CNS disorders could guide the development of targeted treatments for a broad range of patients with these rare diseases.

## 2. Chromatin Plasticity at A Glance

In eukaryotes, genetic information is packaged into chromatin, a highly ordered combination of condensed structures called nucleosomes. It is formed by DNA twisted around octamers of four pairs of histone proteins (namely H2A, H2B, H3, H4) connected to the linker histone H1 with regular intervals of unbound DNA (namely, DNA spacer). This module-based asset is fundamental to fitting DNA within the nuclear space and is pivotal to plasticity. Indeed, chromatin can dynamically change its architecture and functional status in response to appropriate stimuli, thus contributing to cell identity and physiology [8]. In addition, an array of multicomponent complexes orchestrates coordinated deposition (driven by ‘writers’) or removal (driven by ‘erasers’) of chemical moieties, providing chromatin editing [9,10]. In turn, other specific complexes are able to read such marks, thus allowing chromatin to interact with proper RNAs, proteins, or genomic regions and to perform the correct activity in the appropriate spatio-temporal window. Chemical modifications of the chromatin do not alter the genome sequence, are stably inherited, and promptly respond to (extra)cellular signals, hence forming the ‘epi-genome’.

Enzymatic activities engaged in chromatin plasticity include acetylation, citrullination, methylation, phosphorylation, ribosylation, sumoylation, glycosylation, and ubiquitination [11]. Commonly, a combinatorial frame is applied at once, leading to a fascinating dress-code of the chromatin not yet fully dissected. Moreover, epigenetic modifications can be interconnected, reinforcing or dampening in a stepwise fashion the already acquired marks-driven outcomes [12]. The emerging and fascinating role of epigenetic factors in autophagy regulation particularly captured our attention [13]. In this context, advances in next-generation sequencing and epigenome profiling studies have helped us to understand the link between specific epigenetic dysregulation and neurodegenerative disorders [14], thereby identifying the epigenome as a druggable target. However, functional damage to chromatin regulators also results in neurodevelopmental diseases [15]. Generally speaking, a broad spectrum of intellectual disabilities (e.g., autism-spectrum disorders, Down syndrome, fragile X syndrome, Parkinson’s disease) are featured by common alterations of metabolic homeostasis and structural as well as functional mitochondrial anomalies [16,17]. Likely, convergent pathways in genetic pathologies with different causative gene(s) may shed light on common disease mechanisms and put in place similar therapeutic strategies.

### 2.1. DNA Methylation/Hydroxymethylation and Their Readers

DNA-modifying enzymes include DNA methyltransferases (DNMTs) that add a methyl moiety from S-adenosyl methionine (SAM) to 5′-carbon of cytosine, mainly at CpG (cytosine-phospho-guanine dinucleotide)-enriched promoters, or at CpH (where H is A, C or T) intragenic regions, with opposite functional outcomes in regulating gene expression [18].

Contrary to DNMTs, Ten Eleven Translocases (TETs) actively demethylate DNA, through a base excision repair pathway that produces hydroxymethyl-cytosine intermediates [19]. Interestingly, both SAM and TET enzymatic activities are functionally interconnected with mitochondria. SAM, which supplies methyl groups even to histone methyltransferases, is produced in the cytoplasm and is strictly dependent on the folate cycle that takes place in the mitochondria. Similarly, TET-induced demethylation of DNA is directly dependent on the availability of specific metabolites of mitochondria, such as α-ketoglutarate [20].

DNA methylation is then read by a family of methyl-CpG binding proteins, characterized by a methyl binding domain. To date, ten proteins belong to this class, but the most relevant one is MeCP2 [21], which is also able to read the DNA hydroxymethylation. In fact, MeCP2 is a scaffolding protein for many other chromatin builders and takes part in multiple biological processes [22].

Under physiological conditions, MeCP2 forms a transcriptional repressor complex with the co-factor Sin3a, histone deacetylases (HDACs), and CREB (cAMP response element-binding protein) on the methylated DNA, thus inhibiting the expression of neuronal activity-dependent genes like BDNF (brain-derived neurotrophic factor). However, MeCP2 activity itself is variably regulated through extensive post-translational modifications (PTMs) [23]. For example, upon rat brain stimulation (e.g., with NMDA, IGF1, and CRF) the sumoylation of phosphorylated MeCP2 takes place and reinforces its binding to methylated DNA while allowing dissociation of CREB from the repressor complex [24]. In this scenario and in response to activation of the cAMP signaling pathway, CREB is phosphorylated and recruits CBP (CREB binding protein) on chromatin [25] at its own CRE binding sites, thus promoting the expression of many neurodevelopmental genes, including BDNF [24]. 

### 2.2. Histone-Modifying Enzymes

Histone-modifying enzymes constitute a very huge class of chromatin regulators and can be grouped on the basis of the PTMs they catalyze [26]. They can be further classified on the basis of their subcellular localization (i.e., in the cytoplasm and/or in the nucleus). In recent years, more than 500 histone PTMs have been documented because of a consistent amount of targetable histone amino acids within the nucleosome and on free histones. Many studies have focused on acetylation/deacetylation occurring at specific lysines and catalyzed by histone acetyltransferase (HATs) and HDACs, respectively. In the frame of the gene expression control, there are also histone methyltransferases and demethylases that mostly target lysine or arginine residues. Moreover, enzymes can show different specificities and a distinct degree of modification may be achieved (e.g., mono-di-, tri-methylation of the same residue). Simply changing/restoring the histone’s electric charge, its steric conformation or binding capabilities, histone-modifying enzymes strongly impact the strength of DNA-histone or histone-histone interactions and DNA-based activities of cells (e.g., transcription). Noteworthy, each modification can be actively reversed favoring dynamic transition from a more condensed and inactive structure to more relaxed and active chromatin, and vice versa. Furthermore, mounting evidence suggests that many histone-modifying enzymes also regulate the stability, activity, and abundance of a plethora of non-histone substrates, including DNMT1 and MeCP2 [27,28].

### 2.3. ATP-Dependent Chromatin Remodelers

Chromatin remodelers are ATP-depending molecules that shape the nucleosome structure. They are a broad class of multi-subunit complexes, called SWI/SNF, ISWI, INO80, SWR1, and CHD [29]. Exploiting a translocase domain, they modify the direct interaction between DNA and histones of the nucleosome. Therefore, they regulate the DNA accessibility to transcription factors or other DNA-binding proteins [30]. In this scenario, epigenetic readers are central to decoding modification patterns of the epigenome. Indeed, they participate in the regulation of chromatin accessibility to DNA binding players, directly or in cooperation with ATP-dependent structural modulators [31]. Overall, epigenetic marks and chromatin regulators functionally shape the 3D organization of the genome to respond to specific cellular programs through an elegant and fine-tuned regulation.

## 3. Epigenetic Factors-Driven Neurodevelopmental Disorders with Mitochondrial Phenotype and Subcellular Trafficking Implications

Neurodevelopmental disorders are a highly heterogeneous class of cognitive pathologies, including autism spectrum disorders (ASD), intellectual disability (both autosomal and X-linked), and communication and motor disorders that may share many clinical features [32]. Complexity in their clinical manifestation frequently impacts differential diagnosis and prognosis. Neurodevelopmental disorders have been associated with mutations and variants in a plethora of genes, thus implying the existence of shared regulatory molecular networks [33]. Identifying and characterizing the pathogenic mechanisms involved is essential to unlock the secrets of brain functioning, as well as to develop therapies.

In this category of brain disorders, the primary impairment concerns the synaptic performance and/or neural morphology, which resemble those of immature neurons. Nevertheless, an increasing number of causal genes are rather involved in epigenome shaping. Among them, X-linked pathologies represent a compelling paradigm, as the mammalian X chromosome itself is an example of epigenetic-based expression regulation through the X chromosome inactivation process (XCI) and harbors many neural-related genes involved in chromatin disorders [34]. Interestingly, some autosomal-linked CNS disorders share clinical manifestations with X-linked ones, suggesting that other common mechanisms may exist, independently of the chromosomal localization of causative genes. Here we provide some examples of epigenetics regulators, for which convincing evidence exists about their contribution to the neurodevelopmental phenotype, based on the identification of DNA mutations in independent patients and on supporting animal models generated for functional studies (Supplementary Table S1). Specifically, we selected three neurodevelopmental syndromes with annotated alteration of mitochondria quality with the aim to review the contribution of mitochondria dynamics to the onset/progression of such diseases, while highlighting similarities in the molecular processes involved.

Since maturation failure of neurons is another common feature of neurodevelopmental disorders, we also attempted to point out the analogies with age-related neurological faults. Indeed, despite a trademark of neurodegenerative diseases being the loss of neurons and synapses, recent studies revealed a transcriptional signature of immaturity in neurons from the post-mortem prefrontal cortex of patients with Huntington’s disease [35]. In addition, epigenomic dysregulation is increasingly confirmed as playing a significant role in the progression of most common neurodegenerative diseases [35,36,37].

### 3.1. Methylation Reading and Rett Syndrome

MeCP2 is the main genetic cause of the Rett syndrome (RTT, MIM # 312750), a severe postnatal neurodevelopmental and multisystem disorder that in its classical form affects mainly females. Patients with RTT present with acquired postnatal microcephaly, loss of motor, coordination, and communication skills, hand stereotypes, and various peripheral impairments [34]. Before and soon after the identification of the main causative gene of RTT [38] in different cohorts [39], a pathogenetic involvement of mitochondria was already hypothesized, as suggested by brain anatomy, specific clinical signs (e.g., hypotonia [40] and myocardial dysfunctions [41]) together with findings of altered mitochondria morphology in biopsies of patients with RTT [42,43,44]. Subsequently, direct evidence of systemic mitochondrial dysfunctions relative to redox imbalance and oxidative stress has been collected from human tissue/cells and mouse models of RTT [17,45,46]. Altogether these and more recent studies [47] tried to gain our understanding of the causal relationship between mitochondrial alterations and neuronal dysfunction in RTT and to clarify their contribution to the progression of clinical signs. Mitochondria have been proposed as an interesting therapeutic target [41] while placing the need to find out many more metabolism-related biomarkers, other than those related to oxidative stress [48]. In view of these concerns, the metabolic fingerprints of RTT have been deeply documented both in the brain [49,50] and in different tissues outside of it [51].

Alterations in amino acid and carbohydrate metabolism have recently been confirmed in the cortex of severely symptomatic male MeCP2 KO mice [52]. Nonetheless, in the case of MeCP2-targeted gene expression profiling carried out since early development [53,54] and/or in different tissues [55] might be fundamental in distinguishing between genetic-driven primary defects and secondary features provoked by the accumulation of damages. Indeed, MeCP2 can, directly and indirectly, regulate the expression of an array of nuclear genes encoding mitochondrial proteins (e.g., components of Complex I and III, [56,57]) or factors that support mitochondrial bioenergetics and dynamics [58,59,60].

### 3.2. Rett-like Phenotypes Not Associated with MeCP2 Mutations

Apart from MeCP2, a causality link was initially found between atypical RTT cases and other genes. The MeCP2 duplication syndrome has also been described (MIM # 300815). However, these intellectual disabilities are now classified as clinical disorders distinct from RTT. For example, Cyclin-dependent kinase-like-5 (CDKL5) deficiency disorder (CDD, # 300672) is a severe neurodegenerative disease with early-onset epileptic seizures [61]. Of note, among CDKL5 substrates there is MAP1S, a microtubule-associated protein involved in autophagy [62].

In addition, mutations in the nuclear transcription factor Foxg1 can dictate RTT-like phenotype during forebrain development by virtue of its double localization in the nucleus and in mitochondria [63]. A role of Foxg1 in autophagy is emerging alike [64].

On the other hand, one patient with epileptic encephalopathies (# 308350) and clinical features of Rett carries a mutation in the STXBP1 gene [65] encoding a syntaxin binding protein. Of note, it was found that a common feature of epileptic encephalopathies could be neurodegeneration rather than epilepsy-related regression [66].

### 3.3. Histone Ubiquitination Writing and Mental Retardation X-Linked Syndromic Turner-Type

Mutation in the Huwe1 gene causes Mental Retardation X-linked Syndromic Turner-type (MRXST, # 309590) that shares with RTT a wide range of symptoms and the inheritance mode. Indeed, they are both X-linked diseases, belonging to intellectual disorders with variable penetrance and are mainly caused by de novo mutations in chromatin regulators.

As in Rett syndrome, patients with MRXST present with global developmental and speech delay (or absence of speech), microcephaly, epilepsy, hand stereotypies, autistic features, and motor impairments. Alike MeCP2 [67], the Huwe1 gene is dose-sensitive, as it has been associated with X-related non-syndromic mental retardation due to microduplication in the genomic regions where it is embedded (Xp11.22) [68].

Impaired response to oxidative stress has already been documented in lymphoblastoid cells of a patient with Huwe1 mutation and genomic instability [69]. In normal conditions, Huwe1 triggers DNA damage response (DDR) through histone H1 ubiquitylation [70]. At the same time, DDR is an extra-mitochondrial signal that invokes mitophagy to cope with damage and fix it [71]. DDR takes in place also the nuclear epigenetic factor SIRT1, a stress-response, and NAD+ -dependent histone deacetylase that has been shown to sustain regional specific cognitive function and synaptic plasticity [72]. Notably, MeCP2 is one of the SIRT1 non-histone-substrates and its binding capacity to DNA depends on SIRT1-driven deacetylation [28].

### 3.4. Histone Acetylation Writing and KANSL1/Koolene De Vries Syndrome

Mutation in KAT8 regulatory NSL complex unit 1 (KANSL1) [73] or 17q21.31 microdeletion encompassing KANSL1 cause Koolen-de Vries syndrome (KdVS, OMIM #610443). Patients with KdVS show variable developmental delay, intellectual disability, defective language development, hypotonia, epilepsy, facial dysmorphism, and multisystemic congenital malformations with heart failure. NSL1 is a scaffold complex that assists the histone acetyltransferase KAT8 (also known as MOF) to trigger transcription activation of target genes. Among them, there are autophagy-related genes (ATG) that soon after histone acetylation-mediated induction are then downregulated to prevent prolonged autophagy [74].

As already mentioned for the previous chromatin regulators, KANSL1 is also a dosage-sensitive gene, and its duplications are associated with ASD. Heterozygous Kansl1 mutant mice show hippocampal dysfunctions, likely associated with the reduction of neuronal spine density, whereas no signs of neuronal loss have been noticed [75], as already said for the previous chromatin diseases. Moreover, as oxidative stress increases abnormal and damaged autophagosomes and mitochondria accumulate in brain cells of mutant mice [76]. Again, leveraging on those considerations the parallelism with RTT and MRXST is intriguingly underpinned.

### 3.5. Some Epigenomic Outcomes and Mitochondria Meet on the Ubiquitin Ligase Route of Mitophagy: A Matter of Clearance

Recent studies outlined the essential role of the nucleus in autophagy regulation. Alongside a direct control of the expression of autophagy and lysosome genes [77], the involvement of epigenetic factors is likewise being documented [13]. Epigenetics and autophagy are both highly regulated multi-layered processes, fundamental in sensing and transducing environmental signals. Accordingly, the connection of epigenetics with autophagy is not surprising. In particular, the AMPK (adenosine monophosphate-activated protein kinase) signaling—in early steps of autophagy—links energy balance and nutrient metabolism with the subcellular localization, substrates/interactors accessibility, and the activity of DNMTs and histone-modifying enzymes. It also directly phosphorylates specific histones and transcription factors. Similarly, the amino acids availability influences the balance between protein translation and autophagy through the mTORC1 (mammalian target of rapamycin complex 1) signaling. In turn, mTOR activity influences histone H4 lysine 16 acetylation (H4K16ac) and ATG proteins level by modulating the equilibrium between specific HATs and HDACs, such as KAT8 and SIRT1 [74] respectively. Of note, sirtuins abundance is negatively correlated to their activity and to the energy homeostasis of the cell [78]. Correspondingly, the post-mortem prefrontal cortex of patients with PD, characterized by mitochondrial respiratory complex-I deficiency, exhibits altered NAD metabolism and histone hyperacetylation [37]. What’s more, such histone mark seems to be decoupled from the expected gene activation, especially among nuclear-encoded mitochondrial genes, thus probably impacting mitochondria bioenergetics and dynamics.

Notably, clonal cell cultures that carry truncated forms of the human MeCP2 specifically exhibited H4K16ac hyperacetylation (H4K16ac) [79] but the molecular mechanisms that sustain the dysregulation have not been fully explored. Nonetheless, a component of the mitochondrial complex I, encoded by mitochondrial DNA was found significantly down-regulated in late symptomatic MeCP2 KO mouse brains [56].

**Figure 1:**
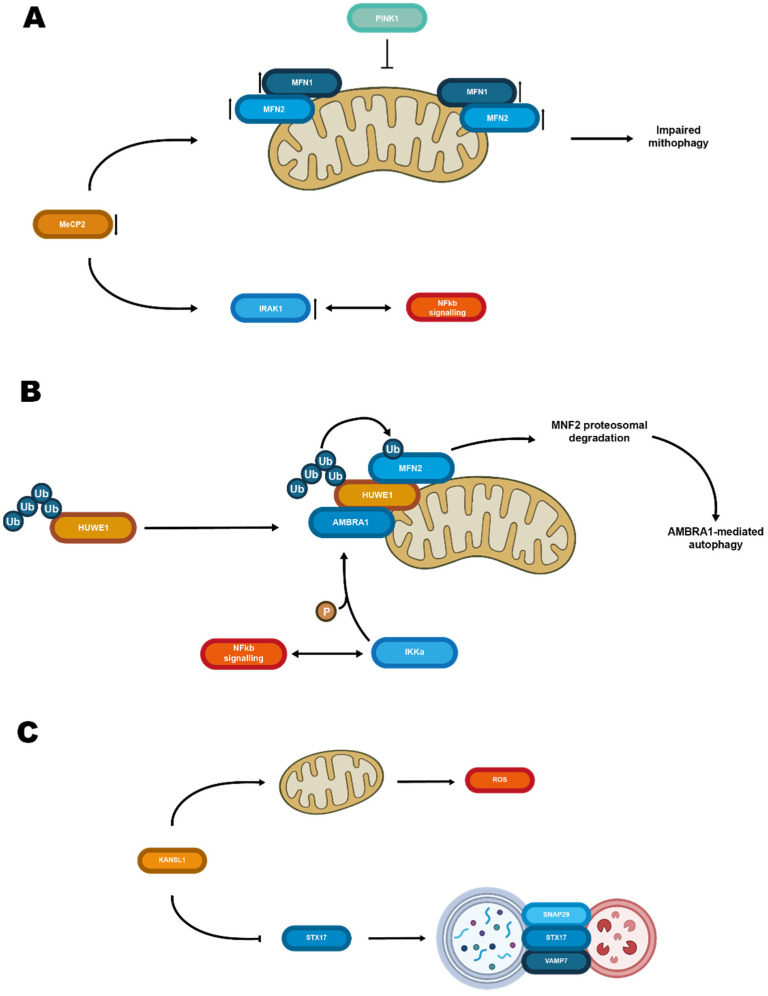
Schematic representation of chromatin remodeling proteins acting on cytosolic elements. (**A**) Mutations or downregulation of MeCP2 results in an increase of Mfn1 and Mfn2 on the mitochondrial membrane impairing mitophagy. Moreover, it also upregulates IRAK1, possibly interfering with NFkb signaling, which results in further impaired neurologic phenotypes. (**B**) Mutations of Huwe1 result in impaired ubiquitination and proteasomal degradation of Mfn2 with a consequent decrease in autophagy. (**C**) Mutations of KANSL1 are associated with mitochondrial damage and ROS production. Suppression of STX17 expression limits autophagosome-lysosome fusion resulting in reduced autophagy.

Defective mitochondrial biogenesis has also been reported in cells derived from RTT patients [58] and in the cerebellum of pre-symptomatic MeCP2 KO mice [80]. RTT fibroblasts display defects in mitochondrial fusion/fission dynamics, likely associated with compromised mitophagic flux [81]. In basal conditions, the (ubiquitin E3 ligase) Parkin protein is less abundant in the RTT fibroblasts when compared to healthy cells, whereas mitofusins Mfn1 and Mfn2 proteins are more enriched in mitochondrial subfractions of the same cell type. Upon in vitro induction of mitochondrial damage, the mitochondrial PTEN-induced kinase 1 (PINK1) fails to translocate to mitochondria (Figure 1A), Parkin completely disappears in induced cells, whereas the kinetics of Mfn2 response is altered [81]. These findings are in line with a model based on an independent cellular system, where PINK1-dependent Parkin-mediated ubiquitination of Mfn2 and mitochondria clearance are proposed to be more efficient when PINK1 and Mfn2 co-localize [82]. Moreover, in the same experimental conditions, RTT fibroblasts are also unable to trigger the caspase-3/7-driven programmed cell death.

Interestingly, Mfn2 is the ubiquitination substrate of other ubiquitin ligases, such as Huwe1. In this case, the mitophagy is triggered by a PINK1/Parkin-independent pathway, that exploits autophagy/beclin-1 regulator-1 (AMBRA1) [83], in interacting with Huwe1 and inhibitor of kappa-B kinase (IKKalfa) [84] to catalyze AMBRA1 phosphorylation (Figure 1B). In turn, IKK is an IRAK1 interactor that plays an essential role in nuclear transcription factor NF-kappa-B signaling [85]. This pathway has been directly associated with a wide range of cognitive disorders, including intellectual disability and ASDs [86,87]. Of note, IRAK1 upregulation in MeCP2 deficient mouse brain regions correlates with the severity of RTT [88] and leads to aberrant activation of the NF-kappa-B pathway [89]. On the other hand, dietary supplementation with vitamin D, which is deficient in RTT cells [90], rescues the NF-kappa-B pathway, ameliorates neuronal morphology of male and female mouse models of RTT, and extends the lifespan of male MeCP2 null mice [91]. Among its pleiotropic effects, Vitamin D exerts a neuroprotective effect in a mouse model of Parkinson’s, inducing autophagy [92]; moreover, it suppresses mitochondrial complex I [93] and activates PINK1/PARKIN-dependent mitophagy to limit oxidative stress, while promoting damaged mitochondria clearance [94]. That said, it would be interesting to verify if vitamin D supplementation rescues PINK1/Parkin pathway in RTT cells and to explore mitophagy as well as the NF-kappa-B pathway in Huwe1-mutated patients.

Notably, among KANSL1 downstream nuclear targets are STX17, a key player in SNARE-assisted autophagy (see below), and PINK1. Hence, in KANSL1 mutant mice the formation of the SNAP29/STX17/VAMP8 SNARE complex is inhibited and the autophagy/PINK1-mitophagy pathway is impaired (Figure 1C).

In addition, patient-derived neurons display an accumulation of oxidative stress-mediated autophagosome that impairs neuronal activity and synaptic network [95].

## 4. Traffic at A Glance

In recent years, it has become clearer and clearer that, in addition to genetic and epigenetic control, signaling from the tissue environment (mechano-transduction and molecular cues) [96,97,98] and/or from cargo proteins/RNAs in the shuttle, secreted vesicles (exosomes) contribute to the regulation of cell and tissue growth, differentiation and migration [99]. Cell shaping and formation of cellular processes, such as the neuronal growth cone [100], are crucial to sensing (and responding to) such tissue environmental stimuli. In turn, in eukaryotes, all these pathways depend on conserved subcellular trafficking machinery [101] consisting of multi-subunit complexes that regulate membrane docking, tethering, and fusion. When impaired, the aforementioned molecular pathways can result in severe CNS diseases as they mediate biogenesis, fusion/division of membrane compartments (ER, ERGIC; Golgi), and organelles—including mitochondria, as well as endocytosis and exocytosis, autophagy and mitophagy.

Eukaryotic cells exploit macro-autophagy (hereby simply referred to as “autophagy”) to degrade and recycle cellular components, including protein aggregates and dysfunctional organelles. Autophagy relies on autophagosome formation by fusion of the phagosome with lysosomes and degradation of its cargo by lysosomal hydrolases. Since autophagy is crucial for cell response to metabolic stress and maintaining homeostasis, its dysfunctions are associated with many diseases, including cancer, immune disorders, and neurodegeneration [102,103]. Autosomal recessive forms of early-onset Parkinson’s disease are known to be associated with mutations in PINK1 and the E3 ubiquitin ligase Parkin, which have been found to promote mitophagy, a specialized form of autophagy degrading damaged mitochondria [104,105,106]. Mutations in huntingtin have been found to impair both autophagosomes cargo recognition [107] and axonal transport [108] in cell models of Huntington’s disease. Furthermore, mutations in presenilin-1, an enzyme involved in amyloid ß plaques formation, are known to increase lysosomal pH and limit autophagy in familial forms of Alzheimer’s disease [109].

Mechanistically, autophagy is divided into four sequential stages: (i) initiation, (ii) autophagosome formation, (iii) autophagosome maturation, and (iv) lysosomal degradation [110]. The former two stages are mediated by a series of kinase complexes and autophagy-related proteins (ATGs) that senses alteration in cell homeostasis and initiate the process. In contrast, the two final stages rely on acidic lysosomal hydrolases that maintain the digestive activity of lysosomes and on a set of membrane-associated proteins mediating autophagosome trafficking and autophagosome-lysosome fusion [111]. Specifically, the process is temporally and spatially controlled by Soluble N-ethylmaleimide-sensitive factor (NSF) Attachment protein Receptor (SNARE) proteins, such as the Q_a_-SNARE STX17, the Q_bc_-SNARE SNAP29, and the R-SNAREs/vesicle-associated membrane proteins (VAMPs) VAMP7 and VAMP8, several tethering factors, such as the HOPS complex, ATG14, and EPG5, and related regulatory proteins (e.g., Rab7, RILP, PLEKHM, BRUCE, and Pacer) [112,113,114,115,116,117,118].

## 5. SNARE and Tethering Factors

SNARE proteins mediate vesicle trafficking inside the cell. While displaying a broad structural variability, they are characterized by at least one SNARE motif, a functionally conserved coiled-coil region mainly composed of hydrophobic residues arranged in a heptadic register. Such register is interrupted at the so-called “zero layer” by a conserved R or Q residue regulating SNARE proper interaction during membrane fusion [119]. SNAREs provide much of the mechanical energy and specificity required during membrane fusion. Specifically, one R-SNARE on a vesicle surface and three Q-SNAREs on the opposite target membrane zip together, from the membrane distal regions towards the proximal ones, to form the trans-SNARE complex (four-helix bundle) [120]. The energy released during the assembly brings membranes close together and drives their fusion [121]. SNARE proteins activity is strictly regulated by post-translational modification and may be further regulated by their N-terminal region, which in short R-SNAREs/VAMPs or synaptobrevins may consist of a short unstructured peptide of 10–30 residues, while in long VAMPs or longins is a globular domain of 100–150 residues. In VAMP7 and other longins, such longin domain can fold back onto the SNARE motif to prevent four-helix bundle formation [122]. With the other two prototypical longins, Ykt6 and Sec22 [123], VAMP7 plays a pivotal role in autophagosome biogenesis, maturation, and fusion to lysosomes [124,125]. Particularly, regulation of autophagy by VAMP7 is crucial to maintain mitochondrial homeostasis [114] as well as to secretory endoplasmic reticulum (ER)-autophagy, i.e., reticulophagy/ER-phagy (SERP), which when inhibited results in a severe impairment of neurite outgrowth [126]. VAMP7 knockout (KO) neuronal cells show impaired neurite growth and reduced secretion of Reticulon 3 (RTN3), which is an ER-phagy related protein [127]. The long isoform of RTN3 (RTN3L) is an effector of Rab32, a small GTPase that (i) controls targeting mitochondria-ER contact sites (MERCS), thus influencing the composition of the mitochondria-associated membrane (MAM) and (ii) regulates mitochondrial membrane dynamics [128].

Phosphorylation of Ykt6 modulates its interactions in a Parkinson’s disease model, suggesting a potential involvement of this longin also in PD [129]. Indeed, Parkinson’s disease α-synuclein perturbs the physiological response to lysosomal stress by impeding Ykt6 [130]. Intriguingly, in the mouse brain cortex, Ykt6 was found to interact—together with a number of mitochondrial proteins—with Huntingtin [131].

### 5.1. Syntaxin17

Syntaxin17 (STX17) is one of the six ancestral Q-SNAREs, and it has been associated with autophagosome-lysosome fusion and mitochondrial division [132,133]. It contains an N-terminal H_abc_ domain, a Q_a_-SNARE motif, and two hairpin-forming tandem transmembrane domains. Although inactive STX17 is an ER-resident SNARE, its acquisition by autophagosomes marks the progression of nascent autophagosomes toward autophagosome-lysosome fusion [134]. However, STX17 knockout mammalian cells partially retain autophagosome-lysosome fusion, which is mediated by longin Ykt6 [135,136].

**Figure 2:**
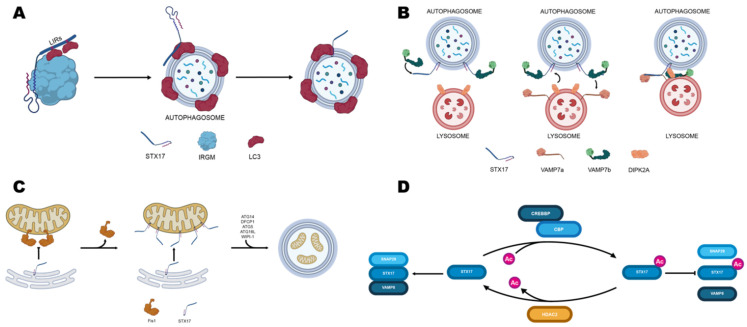
Role of STX17 in autophagy and mithophagy. (**A**) Schematic model for STX17 recruitment to autophagosomes, as reported by Kumar and co-workers. Image was adapted from reference [137]. (**B**) Schematic model for STX17 interaction with VAMP7a, VAMP7b, and DPIK2a in mediating endosome-lysosome fusion as proposed by Tian and co-workers [133] (**C**) Schematic model of STX17-induced mitophagy upon mitochondrial fission 1 protein knockout. Image was adapted from reference [138]. (**D**) Interplay between histone acetylase and deacetylase regulates STX17 ability to mediate autophagosome fusion with lysosomes.

Recruitment of STX17 onto autophagosomes depends on IRGM (Immunity Related GTPase M), which binds it to its C-terminal region. In turn, IRGM interacts directly with members of the mammalian ATG8 family (mATG8s) to form the autophagosome recognition particle (ARP), which delivers STX17 to the autophagosomal membrane [137]. To prevent premature engagement in trans-SNARE complexes, mATG8s are also predicted to bind STX17 by its LC3 interacting region (LIR) located within the SNARE motif (Figure 2A) [137].

Moreover, acetylation of the SNARE motif by the histone acetyltransferase CREBBP/CBP (CREB binding protein) further prevents four-helix bundle formation by steric hindrance. Deacetylation by histone deacetylase HDAC2 promoted by CREBBP inactivation favors STX17 interaction with SNAP29 and the homotypic fusion and protein sorting (HOPS) complex (Figure 2D). This interaction, in turn, promotes the formation of the STX17-SNAP29-VAMP8 SNARE complex and the autophagosomal recruitment of HOPS, which eventually leads to autophagosome-lysosome fusion [112].

STX17 has also been found to compete with two splice variants of VAMP7 [139] VAMP7a, VAMP7b, and DIPK2a for endosome-lysosome fusion (Figure 2B). Coimmunoprecipitation assays showed that STX17 could bind either VAMP7a and VAMP7b C-terminal region. Particularly, VAMP7b binding to STX17 is an alternative to that of VAMP7a and results in reduced lysosome-autophagosome fusion. The same study found that DIPK2a (divergent protein kinase domain 2A) inhibits the interaction between STX17 and VAMP7b, promoting binding between STX17 and VAMP7a and, thus, endosome-lysosome fusion [133].

It has been shown that STX17 can translocate from MERCS to mitochondria, where it promotes mitophagy [138,140]. Xian and co-workers found that Fis1, a protein located in the mitochondria outer membrane, acts as a gatekeeper, governing the traffic of STX17 from ER to mitochondria. Moreover, they found that ablation of Fis1 primes the over-transportation of STX17 to mitochondria, subsequently recruiting ATG14 and thus promoting mitophagy in a PINK1/Parkin independent fashion (Figure 2C) [138].

Given its central role in directing trafficking during mitophagy, STX17 dysfunctions have been linked to processes related to Parkinson’s disease.

STX17 has been shown to interact with PGAM5, a phosphoglycerate mutase family member, which exhibits protein phosphatase activity. Deficiencies of PGAM5 have been shown to cause Parkinson’s-like movement disorder and resistance to metabolic stress [141,142,143]. Although involved in many processes, PGAM5 is required for dephosphorylation of the mitophagy receptor FUNDC1, which, in turn, mediates the interaction between mitochondria and autophagosome-bound LC3 [144]. Sugo and co-workers found PGAM5 and STX17 to be strictly related, as depletion of STX17 in HEK293T cells prevented the interaction of PGAM5 and FUNDC1 upon treatment with the uncoupling agent carbonyl cyanide 3-chlorophenylhydrazone (CCCP) [145].

Morales and co-workers described trans-mitophagy in a mouse model of Parkinson’s disease [146]. They found mitochondria accumulating dopaminergic terminals are engulfed in spheroids upon axon fragmentation. Such spheroids consist of saccular structures of 2–9 µm diameter and are positive for several proteins involved in PINK/parkin-dependent mitophagy, including PINK1, Parkin, Ubiquitin, AMBRA1, and LC3, indicating the presence of nascent autophagosomes. However, they found that autophagosome maturation is prevented by the absence in spheroids of STX17, LAMP1, and LAMP2. Spheroids are transferred to surrounding astrocyte processes to complete autophagosomal maturation, as it has been shown that astrocytes express high quantities of STX17, LAMP1, LAMP2, and Bcl2L13. Overall, Morales’s work suggests mitophagy of degenerating terminals starts in spheroids and finishes in the surrounding astrocytes, and thus new scenario for the pathogenesis of PD since a failure in trans-mitophagy could be the basis of the acceleration of the dopaminergic degeneration, which occurs typically at the beginning of Parkinson’s disease.

### 5.2. SNAP29

SNAP29 is a Q_bc_ SNARE interacting with STX17 and VAMP8 in mediating autophagosome-lysosome fusion (4–6) and with STX17 and VAMP7 in mediating mitochondrial-derived vesicles endolysosome fusion [147]. Mutations in SNAP29 cause Cerebral Dysgenesis, Neuropathy, Ichthyosis, and Keratoderma (CEDNIK), a rare congenital neurocutaneous syndrome associated with short life expectancy, whose pathogenesis is unclear [148]. By characterizing a zebrafish SNAP29_K164X mutant line, it was found that mitochondria were included in a double membrane, suggesting potential alterations in membrane trafficking during mitophagy [149].

### 5.3. Tethering Factors

Important elements in multi-subunit complexes mediating tethering in eukaryotic cells are vacuolar protein sorting (VPS) elements. VPS39 is one out of the six subunits of the HOPS complex, which represents the structural bridge necessary for the fusion of late endosomes and autophagosomes with lysosomes [150]. Mutations in VPS39, a proven de novo schizophrenic gene in chromosomal locus 15q15, have also been suggested to be involved in periodic catatonia, by disrupting lysosome-mitochondria tethering and transport of lipids and calcium through membrane contact sites [151]. Mutations in VPS11, damaging the autophagic and lysosomal pathways, are the probable genetic cause of a novel form of generalized dystonia [152].

In humans, there are four VPS13 family members and mutations in hVPS13A/B genes that are linked to rare neurodegenerative disorders chorea- acanthocytosis (hVPS13A) and Cohen syndrome (hVPS13B), while those in VPS13C/D predispose to early-onset into Parkinson disease (hVPS13C) and lead to ataxia/spastic paraplegia (hVPS13D). VPS13 seems to actively participate in an exchange of the lipids between membranes of organelles in membrane contact sites and impaired VPS13 function results in changes in intracellular protein trafficking between Golgi apparatus, plasma membrane, and endosomes, mitochondria functioning and cytoskeleton organization [153]. Recently, it has been found that ataxia/spastic paraplegia-associated isoform VPS13D is a key regulator of MERCS and VPS13D suppression leads to severe defects in mitochondrial morphology, mitochondrial cellular distribution, and mitochondrial DNA synthesis [154]. Lastly, it has been shown that deficiency or mutation of Vacuolar protein sorting-35 (VPS35), a retromer component for endosomal trafficking, has been linked to familial Parkinson’s disease depending on Mfn2 degradation and mitochondrial fragmentation [155,156].

## 6. Rabs

Ras-like proteins from brain (Rabs) are the broadest branch of the GTPase superfamily, comprising at least 66 members in the human genome [157] and act as molecular signatures for intracellular vesicles, influencing their biogenesis and function [158,159]. All Rab proteins contain a Ras-like small GTPase domain and are reversibly anchored to membranes by geranyl-geranyl groups attached to their C-termini. By attaching to membranes, they can recruit many effectors for membrane tethering, fusion, sorting, and signaling, thus conferring functional plasticity to vesicles [159,160]. Being GTPases, they alternate between a GTP-bound ‘on’ form and a GDP-bound ‘off’ form, promoting the docking of transport vesicles with specific target membranes. 

Rabs intrinsic GTPase activity can be enhanced by GAPs (GTPase-activating proteins) which facilitate GTP hydrolysis. In contrast, GDP exchange with GTP is favored by guanosine exchange factors (GEFs). Each Rab, GAP, and GEF has a strict specificity for a restricted number of targets, favoring precise combinatorial control of vesicular trafficking [161].

### 6.1. Rab5

**Figure 3:**
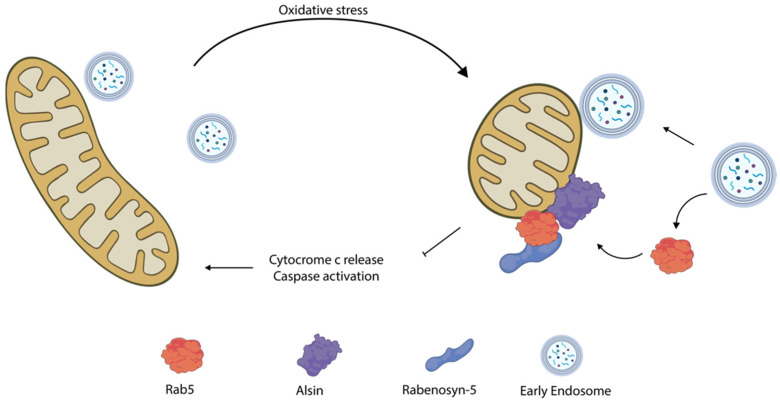
Schematic model of the role of Rab5 and Alsin during mitochondria oxidative stress. Image was adapted from reference [162].

Many Rabs have been found to be targets of proteins mutated in ALS, MS, PD, and HD. For instance, Hsu e co-workers described a cytoprotective mechanism during oxidative stress entailing the translocation of Rab5 from early endosomes to mitochondria (Figure 3) [162]. The authors found that, upon oxidative stress, Rab5 along with his GEFs Alsin and Rabenosyn-5 are rapidly recruited onto mitochondria within minutes from damage, preceding activation of any components of the mitophagic pathway. Here, the Rab5 machinery forms contact sites with early endosomes and mediate the transfer of lipid and metabolites to patch up mitochondrial wounds. In this process, the stress response triggers the solubilization of Rab5 from early endosomes. However, its activation of mitochondria depends on the Rab5 GEF Alsin, which translocates from the cytosol to mitochondria upon stress conditions [163,164]. 

Defects in Alsin involve a deficiency in Rab5 recruitment to mitochondria, thereby leading to a decline in protection from ROS and oxidative stress. Although the involvement of this process in ALS etiology is still unclear, the authors postulate that the oxidative stress it involves might be relevant to the progression of the disease.

### 6.2. Rab7

Kim and co-workers recently showed that Rab7 might also be involved in Parkinson’s disease etiology by prolonging mitochondria-lysosome (M-L) contact sites [165]. They obtained neurons from a Parkinson’s disease patient harboring mutant GBA1, a lysosomal enzyme catalyzing the hydrolysis of glucosylceramide, and the greatest genetic risk factor for PD [166]. They found that loss of lysosomal GBA1 activity resulted in an increased percentage of active GTP-bound lysosomal Rab7, which directly mediates M–L contact tethering. At the same time, they also observed a decrease in TBC1D15 (a Rab7 GAP) responsible for M–L contact untethering. The resulting altered axonal distribution of mitochondria and decreased ATP levels were partially rescued by increasing TBC1D15 expression, indicating Rab7 activity and regulation might have a central role in determining M-L contact stability.

**Figure 4:**
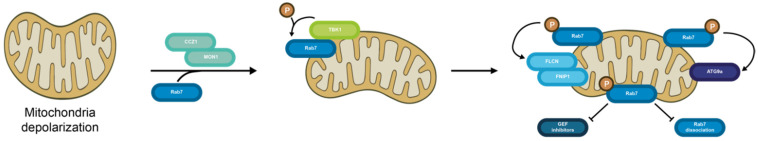
Effects mediated by Rab7 upon mitochondria depolarization. Rab7 phosphorylation on serine 72 by the mitochondrial outer membrane kinase TBK1 triggers recruitment of FLCN-FNIP and ATG9a. Moreover, loss of interactors with GDP dissociation inhibitors, and an increase in Rab7 presence in mitochondria membrane, favors mitophagy.

Moreover, upon mitochondrial depolarization, Rab7 is recruited to mitochondria (Figure 4) through the CCZ1-MON1–dependent pathway, and phosphorylated on S72 by TBK1, a mitochondrial outer membrane kinase that associates to mitophagy cargo receptors during PINK/PARKIN mediated mitophagy [167,168,169]. Rab7 S72 is a conserved residue among Rabs, and it is located in its switch II domain, which is involved in GDP/GTP exchange and in determining protein interactors. Thus, Rab7 phosphorylation leads to (i) a decrease in the interaction with GDP dissociation inhibitors, (ii) an increase in the recruitment of the FLCN-FNIP1 complex (Rab GEF not acting on Rab7), and (iii) the recruitment of ATG9a on damaged mitochondria, which, in turn, promotes mitophagy. Moreover, phosphorylation of Rab7A S72 may reduce the rate of removal of Rab7A from membranes in the vicinity of damaged mitochondria until pS72-Rab7A is dephosphorylated, thus making Rab7 a key player in orchestrating mitophagy.

### 6.3. Rab8

Alterations in Rab phosphorylation are common in PD as Loss of function mutations of PINK1 and gain of functions mutations of the Leucine-Rich Repeat Kinase 2 (LRRK2) are known to be among its most common causes [170]. Both proteins can phosphorylate a specific subset of Rab proteins in two conserved but different residue (S111 and T72 respectively), thus impairing the ability of Rabs to interact with GEFs and GAPs. However, the interplay between the two kinases has long been neglected. It was recently found that PINK1-dependent phosphorylation of Rab8 may interact antagonistically with LRRK2 direct phosphorylation [171]. Rab8 is involved in Golgi regulation and vesicle trafficking [172,173], and interacts with the multifunctional adapter protein optineurin (OPTN), forming a complex that helps maintain Golgi morphology and post-Golgi trafficking to the plasma membrane and to lysosomes [174]. Upon starvation, its GEF Rabin8 mediates its translocation in mitochondria, where it has a yet unknown function [175]. PINK1 can indirectly phosphorylate Rab8 in position 111, thus preventing its interaction with GEF Rabin8 [176]. Moreover, LRRK2 is known to phosphorylate Rab8 at Ser 72 locking it in an active state. It was recently shown that PINK1 and LRRK2 regulate Rab8 phosphorylation state in an antagonistic fashion. Specifically, PINK1 mediates phosphorylation of pS111-Rab8 and prevents phosphorylation of pT72-Rab8 by LRRK2 [171]. Given the interplay of PINK1 and LRRK2 in PD and their effect in mediating Rab8 function it is possible that Rab8 may represent a molecular nexus in PD-linked signaling pathway [176].

### 6.4. Rab11

Rab’s mediated trafficking is also altered in Huntington’s disease. Defective Rab11 activation in Htt^140Q/140Q^ mice models of HD [177,178] results in a decrease in membrane recycling of EAAC1, a Na^+^-dependent glutamate transporter [179]. EAAC1 is the main cysteine transporter in neurons and a lack of cysteine results in a decrease in GSH synthesis and in an increase in cell oxidative stress. It has been proposed that impairment of mitochondrial function, which appears in later stages of HD progression, may be the result of a steady buildup of ROS and an increase in oxidative stress from a chronic deficiency in GSH synthesis [180].

### 6.5. Rab32

Rab32 regulates mitochondrial-associated proteins and ER-mitochondria interactions and dynamics by shuttling between ER and mitochondria [181]. Expression of Rab32 is increased in neurons and microglial cells of both human and mouse models of multiple sclerosis (MS). Haile and co-workers found this increase is due to ER stress directly promoting neuronal cell death from a combination of apoptosis and necroptosis, alterations in mitochondrial morphology, and neurite outgrowth [182]. The authors suggested inhibition of Rab32 production could inhibit neurodegeneration in vivo. Moreover, Rab32 is also involved in the direct trafficking of mitochondria around the cell, as overexpression of a constitutively inactive form of Rab32 was shown to slow down the mitochondrial movement towards neurites in SH-SY5Y cells [183]. Since defects in mitochondrial axonal transport are known to be an early sign of neuroinflammation, it is possible that overexpression of Rab32 in MS acts as a global negative factor for neuronal mitochondria [183,184]. In a study aimed to identify novel CpG sites the methylation of which might be correlated to the chronological and biological age, individuals exhibiting methylation levels of the *RAB32* CpG site higher than 10% were observed to be more prone to disability than people with lower levels, providing the first evidence that epigenetic modifications of genes involved in mitochondrial quality control occur over time according to the aging decline [185]. Rab32 and its closest homolog Rab38 interact with the PD-associated LRRK2 kinase, and Rab32 mediates LRRK2 late endosomal transport and sorting in the cell [186]. In addition to the indirect association to PD via LRRK2 interaction, Rab32 (together with another brain expressed Rab, Rab39) has been found to directly be causative for this disease when mutated [187].

Rab32 collaborates with the multi-ligand binding protein megalin (LRP2) in the trafficking of intracrines (angiotensin II, stanniocalcin-1, and TGF-β) from the cell surface to the mitochondria through the retrograde early endosome to Golgi pathway [188]; when impaired, this pathway may contribute to the pathogenesis of two severe, multisystem and neurodevelopmental genetic syndromes of Donai Barrow and Lowe [189].

## 7. Other Proteins

### 7.1. Mitofusins

Mitofusins (Mfn1 and Mfn2) are transmembrane GTPases, embedded in the mitochondria outer membrane, which are required for mitochondria fusion and tethering to the ER [190]. Playing a pivotal role in mitochondrial development, neuronal maturation, and differentiation [191], their mutation of altered expression is involved in CNS diseases. For instance, abnormal expression of Mfn1 and Mfn2 is caused by the excess of intracellular amyloid-beta (Aβ) in Alzheimer’s and Alzheimer’s-like diseases. Additionally, it is thought to cause the altered mitochondrial morphology and damaged mitochondrial structure that is observed in hippocampal neurons in the early stages of the diseases [192]. Mutations of Mfn2 resulting in impaired mitochondrial axonal transport can be causative of Charcot-Marie-Tooth disease (CMT) 2A, characterized by optic atrophy, motor, and sensory neuropathy, and a variety of brain lesions [193,194,195]. Along with the GTPase OPA1, Mfn2 is also known to cause altered mitochondrial functions depending on the mutation of the mitochondrial-expressed Branched Chain α-Keto acid Dehydrogenase Kinase (BCKDK) gene, which has been associated with a dietary-treatable form of autism [196]. Presenilin 2 (PS2) mutations underlie familial Alzheimer’s disease (FAD) by promoting ER-mitochondria coupling only in the presence of Mfn2 [197]. Lastly, in a mouse model of global brain ischemia, Mfn2 was found to play a central role in mitochondrial dysfunction associated with neuronal misfunction or death [198,199].

### 7.2. Other Trafficking Factors

GTPase-driven maturation of the peptidyl transferase center (PTC) is crucial to mitochondrial ribosome biogenesis and a process mediated by several factors, including GTPases GTPBP5, 6, 7, and 10 [200], and complex consisting of mitochondria transcription termination factor MTERF4 and RNA methyltransferase NSUN4 [201]. MTERF4 plays a role in AD syndrome, shifting APP processing from α-to β-cleavage and promoting the amyloidogenic processing of APP [202]. *GTPBP6* was found to be among differentially expressed X-linked genes in an association study of verbal cognition defects in a group of Klinefelter’s Syndrome (KS) individuals [203]. Both *GTPBP6* and *SYBL1* (encoding VAMP7) are involved in cognitive development, and *GTPBP6* expression has been found negatively associated with full-scale intelligent quotient (IQ) under the regulation of the type of X-chromosome inactivation (XCI) pattern [204]. 

VAMP7 mediates the transport of neuronal cell adhesion molecule L1CAM, playing a central role in brain development and neurite outgrowth [205]. Mutations in L1CAM are causative for a wide range of neurological defects referred to as L1 or CRASH syndrome [206,207,208] and it is noteworthy here that proteolytic cleavage of L1CAM by myelin basic protein leads to the generation of a 70 kDa transmembrane L1 fragment that is imported from the cytoplasm into mitochondria and promotes neuronal migration and neuritogenesis [209]. Very recently, the expression of L1CAM and its 70 kDa cleavage product has been found to be reduced in the hippocampus of AD-like model mice and suggested to contribute to the clearance of amyloid-β in AD brain [210].

### 7.3. VAMP Associated Proteins

The vesicle-associated membrane protein (VAMP)-associated protein B (VAPB) is a type II integral transmembrane protein located in the ER which acts as a tether for contact sites between ER and other organelles, including Golgi and mitochondria [211]. Mutations in human VAPB, cause amyotrophic lateral sclerosis (ALS) [212]. By characterizing vap33 null *Drosophila* (*Drosophila* VAPB ortholog) Kamemura and co-workers found broad alterations in mitochondria morphology and localization. Specifically, they found Golgi was displaced from the cell bodies to dendrites and axons in null neurons, whereas mitochondria were abnormally localized in the cell bodies rather than in neurites. These alterations in organelle localization correlate with a steep increase in dendritic defects and in a reduction in the activity of the ubiquitin-proteasome system [213].

## 8. Post Translational Modifiers

### 8.1. HDACs

Histone modifying enzymes have been treated in the first part of this review because of their role as chromatin regulators and involvement in neurodevelopmental disorders; however, HDACs are both chromatin and trafficking regulators, as demonstrated by the use of HDAC inhibitors as therapeutic agents in trafficking relates diseases. Niemann-Pick type C 1 disease (NPC1) is a lysosomal storage disorder in which, impaired lysosomal cholesterol storage leads to protein misregulation, mitochondrial dysfunction, and cell death, resulting in turn in liver damage and neurodegeneration [214]. HDAC inhibitor valproic acid is able to correct the trafficking defects associated with NPC1 and restore cholesterol homeostasis, an effect that is largely driven by reduced HDAC7 expression [215]. In spite of being a “histone” deacetylase, HDAC6 exclusively deacetylates cytoplasmic proteins, such as α-tubulin, and only in pathological conditions does it become abundant in the nucleus, with deleterious consequences for transcription regulation and synapses, eventually leading to neurodegeneration [216]. 

HDAC6 is a key regulator in mitochondrial dynamics and it is involved in mitochondrial dysregulation in peripheral neuropathies; thus, HDAC6 inhibitors are being investigated as potential therapies for multiple peripheral neuropathic disorders [217]. Indeed, HDAC6 inhibitor Tubastatin A can restore tubulin acetylation levels in MeCP2 deficient cells, representing a potentially new therapeutic option for RTT [218]. Moreover, HDAC6 inhibitors can rescue axonal transport of mitochondria in a primary neuronal culture model of the inherited axonopathy Charcot-Marie-Tooth Type 2F [219], and in neurodegenerative disorders linked to mutations of TDP-43, such as amyotrophic lateral sclerosis with frontotemporal dementia [220].

### 8.2. SUMOylation

Post-translational modification (PTM) of both nuclear and cytoplasmic proteins by SUMO conjugation regulates a diverse array of cellular processes, and indeed, altered SUMOylation of synaptic and mitochondrial proteins is observed in a wide range of neurological and neurodegenerative diseases [221]. Impaired SUMO/deSUMOylation of α-synuclein, DJ-1, and parkin proteins may result in mitochondrial dysfunction leading to Parkinson’s disease [222]. Stimuli able to increase the level of MeCP2 SUMOylation may have therapeutic potential against RTT [24]. Finally, Ataxia Telangiectasia is a neurodegenerative disease characterized by defective mitophagy; this in turn can be caused by impaired SUMOylation of mitofusins [223].

### 8.3. Reversible Phosphorylation

It should be mentioned here that reversible phosphorylation is a highly studied PTM extensively involved in the regulation of mitochondrial dynamics and function, and thus, when impaired, it can be causative for a wide range of disorders, including neurodegeneration; however, this topic will not be illustrated in this review to avoid redundancy with several and recently published reviews on this topic, especially when treated with PINK1, LRRK2, and other kinases [224,225,226,227].

## 9. Conclusions

This review aimed to explore the neglected role of nuclear chromatin and subcellular trafficking in mitochondria-related nervous system disorders. In doing so, we showed that nuclear alterations impacting mitochondria are often associated with an abnormal phenotype linked to altered neurodevelopment. Conversely, mitochondria dysfunctions caused by alterations in cytoplasmic elements often result in neurodegenerations. Although these aspects have often been studied as separate topics, emerging evidence suggests a stronger interconnection might exist between epigenetic regulators, cytoplasmic players, and mitochondria in the frame of brain disorders.

**Figure 5:**
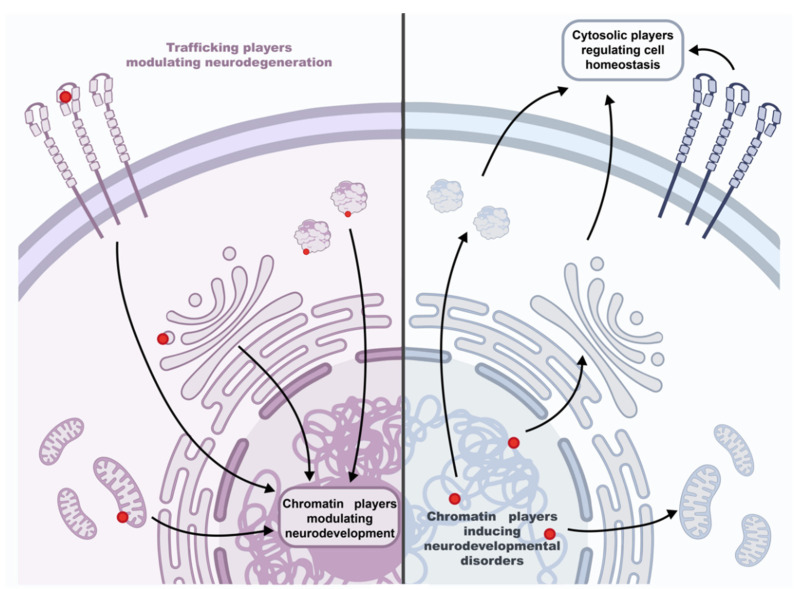
Schematic model of the interplay between nuclear and cytosolic factors in the progression of neurodevelopmental and neurodegenerative disorders. Left panel: mutations (red dots) in trafficking and cytosolic players known to cause neurodegeneration. However, they might also act on chromatin players regulating neuronal development, exacerbating neuronal degeneration. Right panel: chromatin players known to cause neuronal development disorders might also affect cytosolic players involved in maintaining cell homeostasis.

A fascinating mechanistic scenario to further explore in the near future could be the following which is shown in Figure 5.

Mutations in cytoplasmic players involved in organelle trafficking, mitochondrial bioenergetics, and dynamics could also impact the activity of nuclear epigenetic regulators normally leading to neuronal development [14]. In this frame, epigenetic regulators might incorrectly transduce external and internal environmental factors exacerbating neuronal degeneration. In agreement with this hypothesis are the encouraging recovery effects on the progression of neurodegeneration when neurogenesis-promoting epidrugs have been used in preclinical studies. It is the case of amyotrophic lateral sclerosis, a very common adult-onset neurodegenerative disease, for which mitochondria functions can be restored through HDAC inhibitors (reviewed in ref: [228]).

On the other hand, neuronal development is orchestrated by the genetic program, but organelle fusion regulates stem cell fate decisions [229] and promotes the early phase of neuronal differentiation [230]. Thus, genetic mutations that directly impair epigenetic regulators’ functionality could impact the same cytoplasmic players of neurodegeneration, while producing a different outcome. This could be the case of neurodevelopmental disorders related to chromatin without signs of neuronal loss, such as those described here. In this context, pausing neuronal maturation would be a “bankruptcy escape strategy” that cells implement while trying to recover the right nuclear/cytoplasmic communication code. Meanwhile, they start accumulating and coping with the negative effects of the autophagy impasse. This, in turn, would have effects on neuroplasticity and trigger a chain of reactions depending on the neuronal activity (e.g., CREB phosphorylation and CBP recruiting, see also above for the activity of CREB/CBP on SNAREs), that can change the chromatin landscape further, the related-gene expression program and the spatial distribution of cytoplasmic/plasma membrane proteins (e.g., membrane receptors). Correspondingly, mitochondria-targeted interventions have shown to be promising in neurodevelopmental disorders like RTT (reviewed in ref: [59]).

Lastly, the nuclear epigenome—besides being dependent on mitochondria—is also a characteristic of senescence [231]. Therefore, we can tentatively speculate that patients with neurodevelopmental illnesses may exhibit age-related symptoms of neurodegeneration. Of note, elderly patients with RTT show clinical signs of Parkinson’s disease [232], possibly exposing shared common pathways to neurodevelopmental and neurodegenerative diseases. The brain and the heart are highly enriched in mitochondria, therefore mis-autophagy consequences are more pronounced and ready to be detected [233]. The more energy requirement the more devastating effects are expected. Accordingly, neurological disorders manifest with cardiac comorbidity very often, when the impaired player is ubiquitously expressed, or at least it is not neuron specific.

All these biological aspects should be broadly considered in the planning of clinical trials. Most likely, we might combine pharmacological treatments that carefully target both the cytoplasm and the nucleus, possibly in a specific sequential order. Many brain disorders have been shown to be reversible, mirroring the reversibility of epigenetic processes. As a result, further intersectoral efforts should be undertaken to improve the health of affected patients.

## Supplementary Materials

The following supporting information can be downloaded at: https://www.mdpi.com/article/10.3390/biom12050625/s1, Table S1. Epigenetic-related genes that are involved in cognitive disorders.
