# Supplementary material for: Nuclear and Cytoplasmatic Players in Mitochondria-Related CNS Disorders: Chromatin Modifications and Subcellular Trafficking
Source: Biomolecules. 2022 Apr 23;12(5):625. doi: 10.3390/biom12050625 (PMC9138954; doi:10.3390/biom12050625)
Supplement: Supplementary file 1 [file biomolecules-12-00625-s001.zip › biomolecules-1683043-supplementary.pdf]

## Supplementary information to manuscript:

# Nuclear and cytoplasmatic players in mitochondria-related CNS disorders: from chromatin architecture to subcellular trafficking

**Matteo Gasparotto [1], Yi-Shin Lee [2], Alessandra Palazzi [2], Marcella Vacca[2] and Francesco Filippini[1*]**

[1] Synthetic Biology and Biotechnology Unit, Department of Biology, University of Padua, via Ugo Bassi 58/B, 35131 Padua, Italy
[2] Institute of Genetics and Biophysics "A. Buzzati Traverso", CNR, via Pietro Castellino, 111, 80131 Naples, Italy
* Correspondence: francesco.filippini@unipd.it

| Gene symbol | OMIM gene ID | Phenotype | OMIM ID | Epigenetic function | Inheritance | Mitochondria related gene (pubmed*; Mitominer§) | Function related to mitochondria | Cell type in reference | main findings (ref) |
|---|---|---|---|---|---|---|---|---|---|
| **1. Writers** | | | | | | | | | |
| CREBBP (CBP) | 600140 | RubinsteineTaybi syndrome 1 (RSTS1) | 180849 | Histone acetyltransferase (HAT) | AD, de novo | *§ | 1. an essential regulator for the activation of the MSR and in particular the mitochondrial unfolded protein response | MEF | 1. The transcriptional coactivator CBP/p300 is an evolutionarily conserved node that promotes longevity in response to mitochondrial stress |
| | | | | | | | 2. CPB determines mitochondrial dysfunction which leads to impairment of OXPHOX activities and to an increase in ROS production, compromising tissue bioenergetic efficiency. | Human MEF | 2. Induction of mitochondrial dysfunction in patients under cardiopulmonary by-pass: preliminary results |
| DNMT1 | 126375 | Hereditary sensory neuropathy type IE (HSN1E) | 614116 | DNA methylation | AD | * | mtDNA methylation | MEF | DNA methyltransferase 1 mutations and mitochondrial pathology: is mtDNA methylated? |

| | | | | | | | | | |
|---|---|---|---|---|---|---|---|---|---|
| DNMT3B | 602900 | ICF syndrome | 242860 | DNA methylation | AR | * | DNMT3B null hESCs exhibit a disturbed mitochondrial fission and fusion balance and a switch from glycolysis to oxidative phosphorylation | human embryo-nic stem cells | DNMT3B deficiency alters mitochondrial biogenesis and $\alpha$-ketoglutarate levels in human embryonic stem cells |
| EP300 | 602700 | RubinsteineTaybi syndrome 2 (RSTS2) | 613684 | Histone acetyltransferase (HAT) | AD, de novo | * | an essential regulator for the activation of the MSR and in particular the mitochondrial unfolded protein response | MEF | The transcriptional coactivator CBP/p300 is an evolutionarily conserved node that promotes longevity in response to mitochondrial stress |
| EZH2 | 601573 | Weaver syndrome | 277590 | Lysine N-methyltransferase 6 (KMT6A; H3K27me3) | AD, de novo | * | EZH2 knockdown substantially reduced both oxygen consumption rate and calculated reserve capacity. EZH2 promotes the switch from mitochondrial respiration to glycolysis | Glio-blastoma cells | EZH2 promotes metabolic reprogramming in glioblastomas through epigenetic repression of EAF2-HIF1$\alpha$ signaling |
| FTO | 610966 | Growth retardation, developmental delay, coarse facies, and early death | 612938 | RNA demethylation | AR | *§ | FTO down-regulated N6-methyladenosine A levels, decreased mitochondrial content, and increased triglyceride (TG) deposition in hepatocyte. | Hepato-cytes | FTO reduces mitochondria and promotes hepatic fat accumulation through RNA demethylation |
| HUWE1 | 300697 | Mental retardation, X-linked syndromic,Turner type | 300706 | Histone ubiquitination | XL | * | HUWE1 depletion induces reduction of total mitochondriaubiquitylation following AMBRA1 expression which is a relevant mitophagy eceptor | HeLa cells | HUWE1 E3 liase promotes PINK1/PARKIN-independent mitophay by eulating AMBRA1 activation via IKKa |
| KMT2D | 602113 | Kabuki syndrome | 147920 | Histone methylation (MLL2) | AD, de novo | * | reduction of the mitochondrial oxygen consumption rate as well as a reduction of the glycolytic flux in both Kmt2d knockout MEFs and skin fibroblasts of Kabuki patients harboring heterozygous KMT2D pathogenic variants. | Kmt2df/f MEFs | Loss of Function of the Gene Encoding the Histone Methyltransferase KMT2D Leads to Deregulation of Mitochondrial Respiration |

| | | | | | | | | | |
|---|---|---|---|---|---|---|---|---|---|
| UBE2A (Rad6a) | 312180 | X-Linked ID, Nascimento type | 300860 | Histone ubiquitination | XL | * | RAD6A acts as an E2 ubiquitin-conjugating enzyme that, in combination with an E3 ubiquitin ligase such as Parkin, ubiquitinates mitochondrial proteins to facilitate the clearance of dysfunctional mitochondria in cells. Hence, we identify RAD6A as a regulator of Parkin-dependent mitophagy and establish a critical role for RAD6A in maintaining neuronal function. | mRad6a null MEFs | Mutations in the intellectual disability gene Ube2a cause neuronal dysfunction and impair parkin-dependent mitophagy |
| **2. Erasers** | | | | | | | | | |
| HDAC4 | 605314 | Brachydactyly-mental retardation syndrome | 600430 | Histone deacetylase | AD, de novo | * | HDAC inhibitors we examined induced mitochondrial elongation | Hep3B cells | Histone deacetylase inhibitors induce mitochondrial elongation |
| HDAC8 | 300269 | WilsoneTurner syndrome | 309585 | Histone deacetylase | XL | * | HDAC8 inhibits cytotoxicity induced by cobalt and reoxygenation, in part, through suppressing DRP1 expression and mitochondrial fission. | Human renal proximal tubular HK-2 cells | Histone deacetylase 8 protects human proximal tubular epithelial cells from hypoxia-mimetic cobalt- and hypoxia/reoxygenation-induced mitochondrial fission and cytotoxicity |

| | | | | | | | | | |
|---|---|---|---|---|---|---|---|---|---|
| KDM3B | 609373 | Diets-Jongmans Syndrome and Rare Genetic Epilepsy | 618846 | Histone demethylase | AD | * | KDM3B, a histone H3 lysine 9 demethylase, can protect against ferroptosis induced by Erastin, an inhibitor of SLC7A11. (Ferroptosis is a newly discovered form of regulated cell death characterized morphologically by the abnormal mitochondria structure and mechanically by the iron-dependent accumulation of lipid hydroperoxides to lethal concentrations) | Human cell lines (HT-1080) | Histone demethylase KDM3B protects against ferroptosis by upregulating SLC7A11 |
| KDM5C | 314690 | X-linked syndromic mental retardation; Claes-Jensen type | 300534 | Histone demethylase (H3K4 tridemethylase) | XL | * | loss of KDM5C protein mediated glycogen reprogramming was essential for resistance to ROS and ferroptosis. Moreover, KDM5C depletion promoted the anchorage-independent cell growth | Cancer cells | Deficiency of the X-inactivation escaping gene KDM5C in clear cell renal cell carcinoma promotes tumorigenicity by reprogramming glycogen metabolism and inhibiting ferroptosis |
| KDM6A | 300128 | Kabuki syndrome 2 | 300867 | Histone demethylase (H3K27me3/2) | XL | * | genes involved in mitochondrial oxidative phosphorylation, including Cox5a, Cox7a and Cox8b, were increased in the brown adipose tissue of Kdm6aF/Y mice(Myeloid-specific Kdm6a knockout in Kdm6aF/Y) | adipose tissue (mouse) | Kdm6a suppresses the alternative activation of macrophages and impairs energy expenditure in obesity |
| PHF8 | 300560 | Siderius X-Linked Mental Retardation Syndrome | 300263 | Histone demethylase(H4K20 me1, H3K9me1/me2) | XL | * | PHF8 and JMJD3 regulate mitochondrial unfolded protein response genes by removing repressive H3K27 methylation marks from their coding regions. | Hypo-thalamus of the indicated BXD mouse strains | Two Conserved Histone Demethylases Regulate Mitochondrial Stress-Induced Longevity |
| **3. Chromatin remodelers (DEAD/H ATPase family)** | | | | | | | | | |

| | | | | | | | | | |
|---|---|---|---|---|---|---|---|---|---|
| ACTB | 102630 | BaraitsereWinter syndrome | 243310 | SWI/SNF, INO80 and ISWI complex | AD, de novo | * | Defects of Mitochondrial Membrane Potential (MMP) Maintenance in Cells Lacking β-Actin | MEFs | In Mitochondria β-Actin Regulates mtDNA Transcription and Is Required for Mitochondrial Quality Control |
| ARID1A | 603024 | Mental retardation, Autosomal dominant 14 | 614607 | DEAD/H ATPase helicase family, SWI/SNF subfamily | AD, de novo | * | ARID1A loss results in increased mitochondrial metabolism and renders ARID1A-mutated cells increasingly and selectively dependent on it. ARID1A loss associated with increase in c-Myc expression and increased mitochondrial number and reduction of their size consistent with a higher mitochondrial cristae/outer membrane ratio. | Ovarian clear cell carcinom a cells | Targeting mitochondrial metabolism in clear cell carcinoma of the ovaries |
| ATRX | 300032 | Alpha thalassemia mental retardation syndrome, X-linked (ATRX) | 301040 | DEAD/H ATPase helicase family resulting in altered coordination of DNA methylation patterns and H3K9me3 binding | XL | * | Expression of MYCN in ATRX-mutant cells leads to metabolic reprogramming and mitochondrial dysfunctio | Neurobla stoma | MYCN amplification and ATRX mutations are incompatible in neuroblastoma |
| SMARCA2 | 600014 | NicolaideseBaraitser syndrome | 601358 | DEAD/H ATPase helicase family, SWI/SNF subfamily | AD, de novo | * | SMARCA4/2 deficiency causes reduced IP3R3 expression leading to impaired Ca2+ transfer from the endoplasmic reticulum to mitochondria required for apoptosis induction | Cancer cell line (H1437 cells) | SMARCA4/2 loss inhibits chemotherapy-induced apoptosis by restricting IP3R3-mediated Ca2+ flux to mitochondria |
| SMARCA4 | 603254 | Mental retardation, Autosomal dominant 16 | 614609 | DEAD/H ATPase helicase family, SWI/SNF subfamily | AD, de novo | * | SMARCA4/2 deficiency causes reduced IP3R3 expression leading to impaired Ca2+ transfer from the endoplasmic reticulum to mitochondria required for apoptosis induction | Cancer cell line (H1437 cells) | SMARCA4/2 loss inhibits chemotherapy-induced apoptosis by restricting IP3R3-mediated Ca2+ flux to mitochondria |

| | | | | | | | | | |
|---|---|---|---|---|---|---|---|---|---|
| SRCAP | 611421 | Floating-Harbor syndrome | 136140 | DEAD/H ATPase helicase family, INO80/SWR subfamily | AD, de novo | * | The SRCAP complex maintains the integrity of mitochondrial morphology, itochondrial oxidative metabolism and respiratory complexes | mice | The SRCAP chromatin remodeling complex promotes oxidative metabolism during prenatal heart development |
| **4. Other readers and chromatin remodelers** | | | | | | | | | |
| ASXL1 | 612990 | BohringeOpitz syndrome | 605039 | PR-DUB complex, histone H2A deubiquitination | AD, de novo | * | provokes dysfunction of HSCs associated with mitochondrial activation, elevated ROS levels, and increased DNA damage. | Vav-Cre ASXL1-MT KI mice | Mutant ASXL1 induces age-related expansion of phenotypic hematopoietic stem cells through activation of Akt/mTOR pathway |
| BCL11B | 606558 | Immunodeficiency 49 and Intellectual Developmental Disorder With Speech Delay, Dysmorphic Facies, And T-Cell Abnormalities | 618092 | transcriptional repressor | AD | * | Bcl11b is a suppressor of apoptosis with knockout cells showing apoptotic pathway activation, mitochondrial membrane potential loss and elevation of BclxL, Caspase 8, and caspase 9. | malignant T cell lines (Jurkat and huT78) | Proteome analysis reveals new mechanisms of Bcl11b-loss driven apoptosis. |
| CHMP1 | 164010 | pontocerbellar hypoplasia 8 | 614961 | Targets polycomb protein BMI tocondensed chromatin | AR | *§ | detected elevated amounts of the peroxisomal PEX14 protein and outer mitochondrial membrane protein VOLTAGE-DEPENDENT ANION CHANNEL (VDAC) in chmp1 | Plant | The Endosomal Protein CHARGED MULTIVESICULAR BODY PROTEIN1 Regulates the Autophagic Turnover of Plastids in Arabidopsis |

| | | | | | | | | | |
|---|---|---|---|---|---|---|---|---|---|
| CTCF | 604167 | ID, microcephaly and growth retardation | 615502 | Chromatin binding factor, insulator | AD, de novo | * | Genome wide expression analysis in Ctcf mutant hearts revealed that genes controlling mitochondrial function and protein production, required for cardiomyocyte maturation, were upregulated. | Cardio-myocyte | CTCF counter-regulates cardiomyocyte development and maturation programs in the embryonic heart |
| EPC2 | 611000 | associated with intellectual disability, seizures, microcephaly,development delay, hypotonia, and behavioral features similar to autism or Angelman syndrome | / | associated with a chromatin repressive complex | AD | * | EPC2 cells also demonstrated decreased mitochondrial ATPB protein expression by immunofluorescence and swollen mitochondria lacking intact cristae by transmission electron microscopy. | human eso-phageal kera-tinocyte cell lines | Autophagy mitigates ethanol-induced mitochondrial dysfunction and oxidative stress in esophageal keratinocytes |
| HCFC1 | 300019 | Mental retardation, X-linked; MRX3/ Methylmalonic acidemia and homocysteinenia (Cobalamin disorder) | 309541 | Found in repressor and activator complexes | XL | * | The MYC–host cell factor (HCF)–1 interaction influences the expression of genes involved in ribosome biogenesis and mitochondrial pathways. | B lymphocyte cell line (Switchable Ramos cells) | MYC regulates ribosome biogenesis and mitochondrial gene expression programs through its interaction with host cell factor–1 |

| | | | | | | | | | |
|---|---|---|---|---|---|---|---|---|---|
| KANSL1 | 612452 | KooleneDe Vries syndrome | 610443 | NSL1 histone acetyltransferase complex | AD, de novo | * | depletion of KAT8/KANSL1 was shown to cause significant downregulation of mitochondrial DNA transcription and translation, and ultimately impaired mitochondrial respiration. Thus, it is also possible that KAT8/KANSL1-dependent modulation of mitochondrial DNA indirectly regulates PINK1 mitochondrial accumulation and subsequent mitophagy. | POE SH-SY5Y cells | Regulation of mitophagy by the NSL complex underlies genetic risk for Parkinson's disease at Chr16q11.2 and on the MAPT H1 allele |
| MECP2 | 300005 | RETT syndrome; RTT | 312750 | Binds to methylated DNA | XL, mainly females | * | Elevated mitochondrial respiration rates and a reduction in coupling were detected in MeCP2-null male mouse brains | Mouse brain | Gene expression analysis exposes mitochondrial abnormalities in a mouse model of Rett syndrome |
| NIPBL | 608667 | Cornelia de Lange syndrome 1; CDLS1 | 122470 | Cohesin complex | AD | * | si-NIPBL-induced apoptosis was activated by the intrinsic mitochondrial-mediated caspase pathway. Finally, the findings of this study showed that knockdown of NIPBL promoted apoptosis of breast cancer cells in vitro, possibly via inhibition of autophagosome-lysosome fusion | breast cancer cells | Downregulation of Cohesin Loading Factor Nipped-B-Like Protein (NIPBL) Induces Cell Cycle Arrest, Apoptosis, and Autophagy of Breast Cancer Cell Lines |
| SATB2 | 608148 | developmental delay, mild to severe intellectual disability, behavioral problems and abnormal craniofacial features, specifically a cleft palate, Glass Syndrome | 612313 | arranging chromatin packing and organization | AD | * | HDAC1/2 play a conserved role to act in conjunction with SATB2 (mammalian ortholog of DVE-1) to mediate mitochondrial homeostasis. | Human samples, HEK293T cells | Histone deacetylase HDA-1 modulates mitochondrial stress response and longevity |

Table S1. Epigenetic-related genes that are involved in cognitive disorders. There were 31 genes selected and distributed over four categories: 1. writers (9 genes), 2. Erasers (6 genes), 3. Chromatin remodelers of the DEAD/H-ATPase family (6 genes), and 4. Other readers and chromatin remodelers (10 genes). Gene names are provided by the official HGCN symbol (HUGO Gene Nomenclature Committee). The column"OMIM Gene ID" was provided according to the OMIM database (Online Mendelian inheritance in Man;http://www.ncbi.nlm.nih.gov/omim/). Two different source were used for collecting mitochondria-related genes including pubmed* and Mitominer§. The gene function associated with mitochondria are listed which were collected from the reference in the last columns.

AR, autosomal recessive; AD, autosomal dominant;XL, X-linked
